# Research on the mechanism of coal wall fragmentation and dust production characteristics in the perspective of differentiated discrete elements

**DOI:** 10.1038/s41598-024-60192-6

**Published:** 2024-04-23

**Authors:** Zhen Li, Deji Jing, Shaocheng Ge, Xiangxi Meng, Di Yang, Shuaishuai Ren

**Affiliations:** 1 College of Safety Science and Engineering, Liaoning Technical University, Fuxin, China; 2 School of Resources and Environmental Engineering, Jilin Institute of Chemical Technology, Jilin, China; 3 Key Laboratory of Mine Thermodynamic Disasters and Control of Ministry of Education, Liaoning Technical University, Fuxin, China; 4 Research Institute of Safety Science and Engineering, Liaoning Technical University, Fuxin, China; 5 Safety and Emergency Management Engineering College, Taiyuan University of Technology, Taiyuan, China

**Keywords:** Discrete element, Header mining face, Roller cut, Coal wall fragmentation, Dust production characteristics, Environmental sciences, Environmental impact

## Abstract

In order to efficiently and accurately control coal dust pollution in coal mining faces, this study addresses the insufficient research on the dust generation mechanism during cutting. Firstly, a similar experimental platform for simulating coal wall cutting with a drum cutter was used to investigate the changes in coal wall fragmentation and dust generation at drum speeds of 35 r/min, 50 r/min, 65 r/min, and 80 r/min. The experimental results revealed that the degree of coal wall fragmentation and dust generation increased with the increase in rotational speed, leading to a wider range of particle size distribution and an increase in the generation of fine dust particles. A 1:1 scale discrete element simulation of coal wall cutting with a drum cutter was conducted based on the experiments. The results indicated that, under the four rotational speeds, the cracks generated during coal wall fracture were predominantly tensile cracks, accounting for over 76% of the total crack count. The total number of cracks increased from 10,600 to 11,200, the number of free single particles increased from 2555 to 2728, and the fragmentation volume increased from 0.021607 to 0.023024 m^3^. The range and degree of coal wall fragmentation increased with the increase in drum speed.

## Introduction

Mechanized coal mining has become the main coal mining process in China. With the increase in mechanization, the intensity and output of coal mining have increased significantly, but it has also caused serious dust pollution problems^1–5^. The most serious dust pollution area in underground coal mines is the coal mining face. The total amount of dust generated in this area is more than 50% of the dust generated in the mine, and roller cutting by the coal mining machine is one of the main dust sources in the coal mining working face, accounting for approximately 70–80%, which seriously affects production safety and the physical and mental health of the personnel working underground^6–10^. Therefore, the efficient and precise control of underground dust has become a major challenge for healthy production in modern mines^11–15^.

At present, many scholars have conducted much research on the mechanism of dust production and the transport law of coal mining working faces^16–20^. Niu Wei^21^ et al. used Fluent software to numerically simulate the dust production process of coal cutting at the header face and compared the analysis with the actual dust production in the mine to design a spray dust reduction device suitable for the site conditions. Su Shilong^22^ et al. used a Sidepak AM520i individual exposure dust meter to measure the concentration of respiratory dust PM2.5 and PM5 at the front and rear roller measurement points of a coal mining machine during downwind and upwind coal cutting at the coal mining face and analysed the causes of dust concentration variation and dust particle size distribution patterns under various conditions. Zihao Xiu^23^ et al. simulated and studied the effects of different tunnel airflow conditions on the wind flow field and dust dispersion pattern of a coal mining working face and obtained the smallest average dust concentration generated in the range of 1500–1600 m^3^/min for the inlet and outlet airflow. Sun Biao^24^ et al. simulated and studied the wind flow and dust distribution at the coal mining face before and after converging into the rotating air flow of the roller and designed a coal mining machine closed atomization dust removal device based on the simulation study results and applied it in the field. Tan Cong^25,26^ et al. simulated and analysed the dust transport law of coal cutting at a coal mining working face and concluded that the wind speed at the coal mining working face, the coal mining machine roller rotation speed and coal wall conditions are important factors affecting the dust concentration at the coal mining working face, determined the best parameters for dust suppression, designed a similar experimental platform for dust diffusion from multiple dust sources at the coal mining face for experimental study, and concluded that the optimal dust control wind speed of this platform is 1.5 m/s and that the optimal water content should be no less than 3.8%. Peng Cai^27^ et al. used CFD-DPM to analyse and study the wind flow distribution and coal dust transport law in coal mining working faces at different air volumes and concluded that the best dust suppression effect was achieved at an air volume of 2600 m^3^/min. Most of the existing studies are macroscale simulations and analyses of the dust pollution problem for a coal mining working face, while there has been little research into the modelling of the microscopic dust production process at the roller cutting coal wall based on discrete element theory.

In view of this, to comprehensively understand the dust production characteristics and fragmentation mechanisms of drum cutting coal walls, and fundamentally reduce the generation of dust in coal mining working faces, purify the underground working environment, and ensure the physical and mental health of workers, this study utilized a drum cutting coal wall similar platform. By sieving different particle sizes of debris, the roughness index and dust mass fraction at different speeds were calculated. Additionally, laser particle size analyzer was used to analyze the particle size distribution of dust, obtaining the variation law of coal wall fragmentation and dust production at different speeds. Based on the discrete element theory, the PFC simulation software^28^ was employed for numerical modeling to simulate the process of coal wall fragmentation and dust production during drum cutting, and to elucidate the microscopic fragmentation and dust production mechanism of coal walls. The aim was to reveal the influence of drum cutting speed on coal wall fragmentation and dust production, and to provide theoretical support for dust control and environmental purification in mines.

## Experimental platform and procedures for drum cutting of coal walls

**Figure 1:**
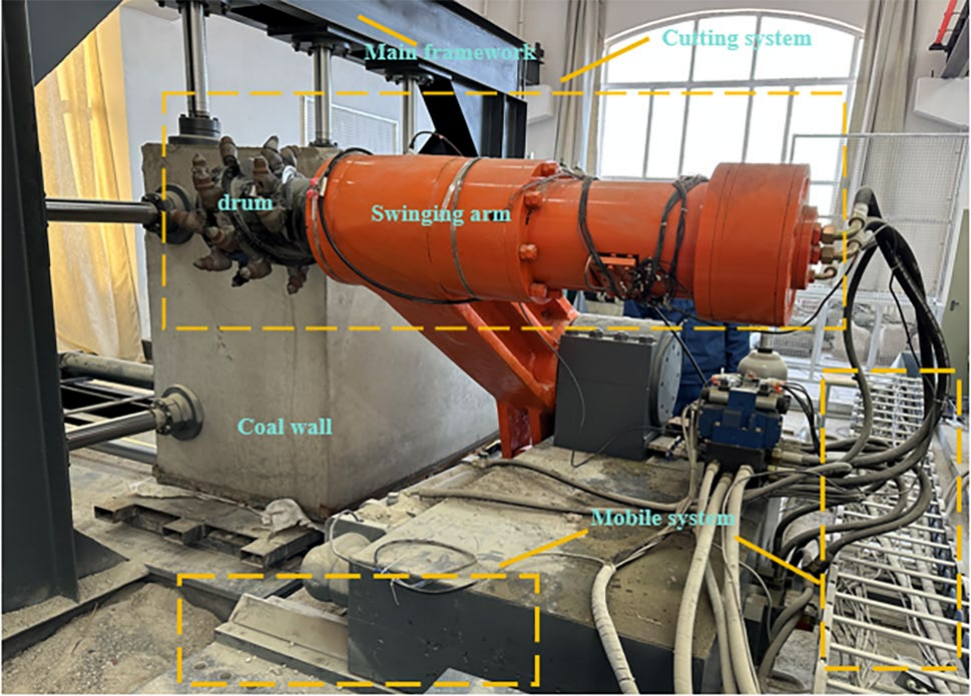
The similar experimental platform of dust production from coal mining machine cut-offs.

The experimental platform shown in Fig. 1 was used to investigate the effects of rotational speed on the degree of coal wall fragmentation and dust generation. The experimental platform primarily consists of the main frame, drum cutting system, moving system, main hydraulic system, and measurement and control system. The specific operational procedures are as follows:

1. The manufactured coal wall is first fixed in the main frame of the cutting test system. Before starting the experiment, the drum, coal wall, and dust collection area are thoroughly cleaned to remove pre-existing deposited dust.
2. The moving system is activated to laterally move the drum to the left side of the coal wall and longitudinally move it to a cutting depth of 25 cm, as shown in Fig. 1.
3. Prior to commencing cutting, a no-load test is conducted on the drum to ensure its proper functioning, and the traction speed of the drum is set to 20 mm/s. The drum cutting experiment at a rotation speed of 35 r/min is initiated, with the drum cutting from left to right until it fully penetrates the coal wall. The diameter of the drum is 35 cm, resulting in a cutting distance of 35 cm. Given the sufficiently large volume of the coal wall, multiple cutting tasks can be completed. Subsequent drum cutting experiments are conducted at rotation speeds of 50 r/min, 65 r/min, and 80 r/min.
4. After each cutting operation, upon allowing the dust to settle in the dust collection area, the debris and dust from the drum, coal wall surface, and dust collection area are collected into containers. An automatic sieving machine is used to sieve the crushed coal debris by particle size, and the mass of all crushed coal debris is measured.
5. A laser particle size analyzer is employed to analyze the particle size distribution of dust particles below 75 μm in diameter, elucidating the distribution pattern of dust particles after crushing.

## Experimental results and analyses

**Figure 2:**
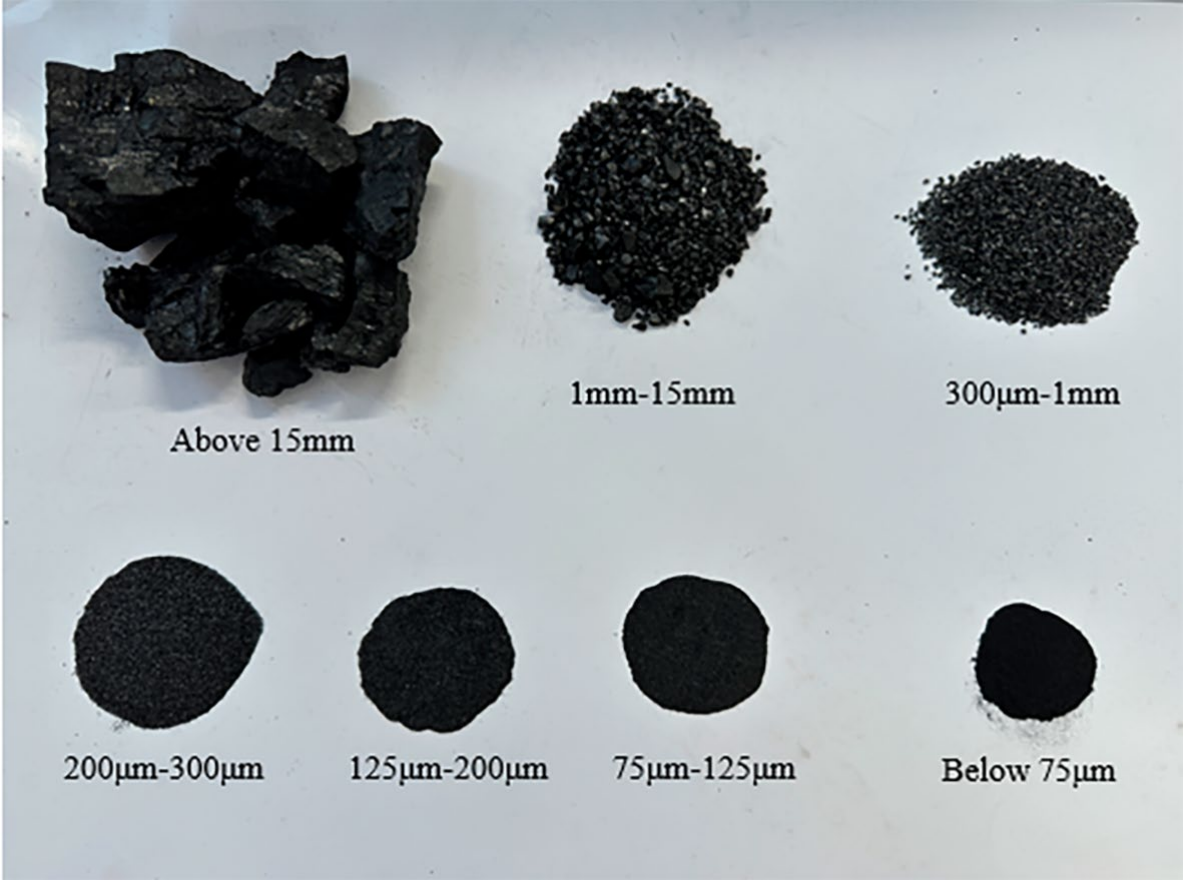
Classification chart of crushed coal chips.

The crushed coal debris collected after cutting at four different drum rotational speeds is sieved using standard sieves with mesh sizes of 15 mm, 18 mesh (1 mm), 60 mesh (300 μm), 80 mesh (200 μm), 120 mesh (125 μm), and 200 mesh (75 μm). Each sieving process is set to a duration of 30 min. Subsequently, the debris of different particle sizes is weighed using an electronic balance, and the corresponding data is recorded. According to the International Organization for Standardization, solid suspended particles with a particle size smaller than 75 μm are defined as dust. The roughness index and the proportion of dust mass below 75 μm at different rotational speeds are calculated, as shown in Table 1. The different particle sizes of the crushed coal debris are illustrated in Fig. 2. The roughness index (*CI*) increases with the increase in the mass of large particle debris generated, indicating a smaller degree of coal wall damage. The formula for calculating the roughness index is as follows:

1 $$CI = \sum {D_{i} }$$

**Table 1:** Particle size analysis of lignite cutting crushing under different rotational speeds.

Particle size	35 r/min	50 r/min	65 r/min	80 r/min
	Cumulative mass percentage (%)	Cumulative mass percentage (%)	Cumulative mass percentage (%)	Cumulative mass percentage (%)
Below 75 μm	100	100	100	100
75 μm ~ 125 μm	99.54	99.48	99.43	99.42
125 μm ~ 200 μm	98.82	98.84	98.64	98.43
200 μm ~ 300 μm	97.59	97.1	97.22	96.54
300 μm ~ 1 mm	95.73	94.92	95.56	94.22
1 mm ~ 15 mm	91.02	88.93	91.2	89.36
Above 15 mm	58.5	53.99	43.6	40.47
Total mass (kg)	22.64	23.33	23.35	25.19
Roughness index *CI*	641.2	633.26	625.65	618.44
Dust mass Percentage (%)	0.46	0.52	0.58	0.67

In the formula, *CI *represents the roughness index, and *D*_*i*_ represents the ratio of the mass of the component debris after sieving to the total mass of the crushed debris.

From the table, it can be observed that under four different rotational speeds, the roughness index of the coal wall after cutting decreased from 641.2 to 618.44, while the proportion of dust mass increased from 0.46 to 0.58%. The roughness index decreases as the drum rotational speed increases, indicating that the degree of crushing increases with higher rotational speed. The proportion of dust mass below 75 μm after cutting of the coal wall increases with the rotational speed, suggesting that dust generation also increases with rotational speed. However, at rotational speeds of 65 and 80 r/min, the proportion of dust mass after coal crushing only slightly increases, indicating that the increase in dust mass proportion tends to level off. This suggests that the rotational speed cannot be increased indefinitely to generate more dust after crushing. Under the same cutting parameters, when the rotational speed reaches a certain level, the dust generation tends to stabilize.

**Figure 3:**
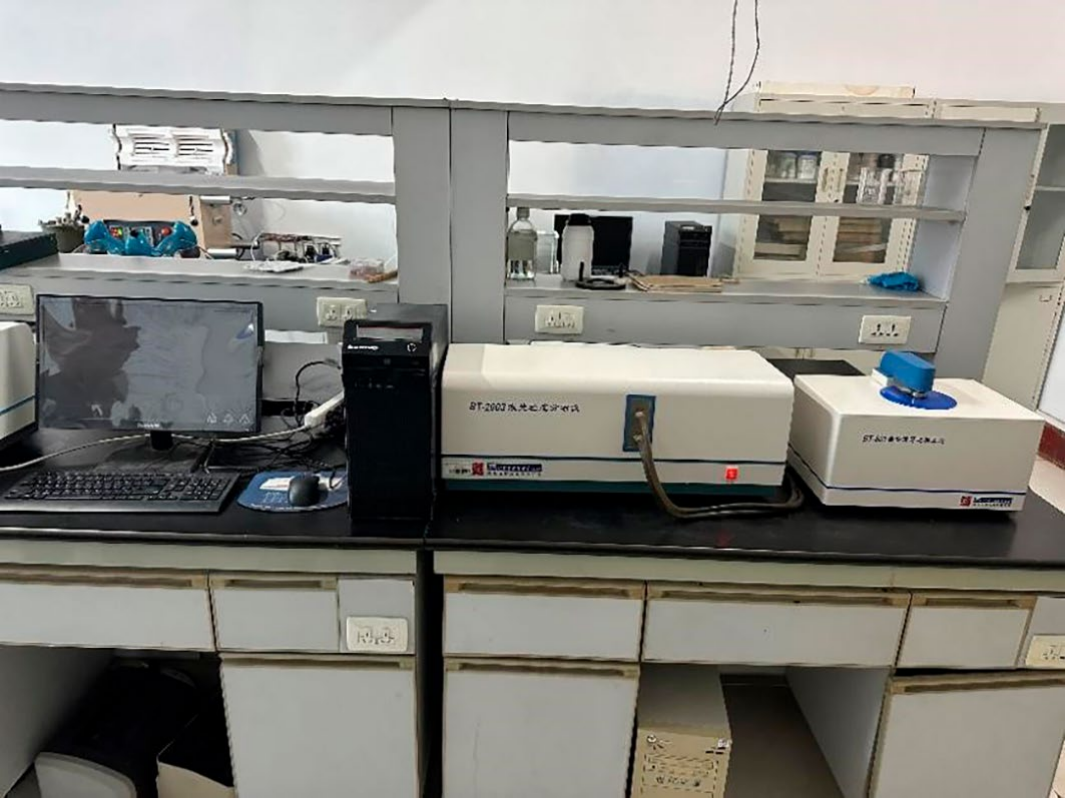
BT-2003 laser particle size analyser.

The cumulative mass rate R of dust below 75 μm generated at four different speeds was measured using the BT-2003 laser particle size analyzer (as shown in Fig. 3), and the measurement results are presented in Table 2.

**Table 2:** Particle size analysis of lignite crushing dust cut by drum under different rotational speeds.

Dust particle size *d* (μm)	35 r/min cumulative mass ratio *R* (%)	50 r/min cumulative mass ratio *R* (%)	65 r/min cumulative mass ratio *R* (%)	80 r/min cumulative mass ratio *R* (%)
Below 1 μm	100	100	100	100
1–15 μm	99.78	99.72	99.51	99.43
15–30 μm	91.31	91.5	90.63	90.31
30 − 45 μm	75.17	75.67	74.85	74.42
45–60 μm	48.63	49.33	48.54	48.86
60–75 μm	31.11	32.08	31.66	31.93

Rosen-Rammler and others derived the widely used Rosen-Rammler distribution function to describe the dust pattern after cutting and fragmentation. This distribution function involves two constants, “*a*” and “*s*”, which are determined based on the dust particle size. The expression for the Rosen-Rammler distribution function is given by:

2 $$\lg \left( {2 - \lg R} \right) = \lg a^{\prime } + s\lg d$$

**Figure 4:**
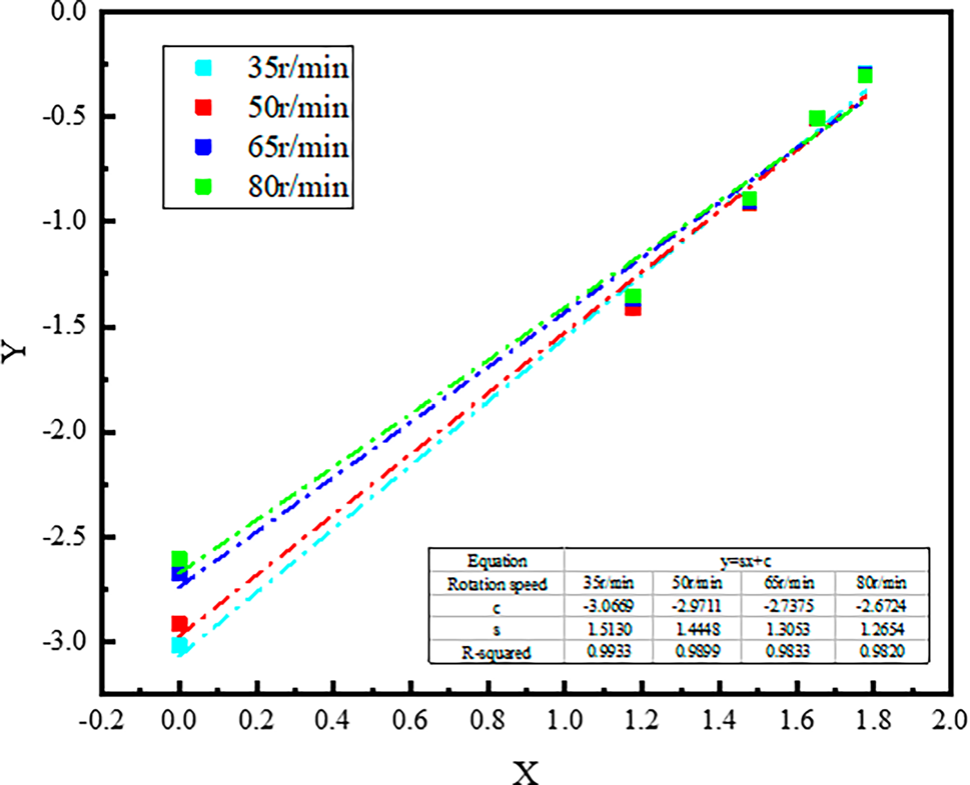
Rosen-Rammler fitting curve of dust after lignite cutting and crushing under different rotational speed conditions.

The dust experimental data were fitted, and the fitting curve should be in the form of y = sx + c, where y = lg(2 − lg*R*), x = lg*d*, c  = lg*a'*,*a* = *a*'ln10 = 2.3*a*'. Using lg*d* as the horizontal axis and lg(2 − lg*R*) as the vertical axis, the Rosen-Rammler fitting graph for coal wall cutting and fragmentation under different rotation conditions is plotted, as shown in Fig. 4.

According to the fitting curve equation, the experimental constants *a* and *s* for the Rosen-Rammler distribution were calculated, as shown in Table 3. The value of *s* represents the slope of the fitted line, indicating the uniformity of particle size, referred to as the distribution index. A larger *s* value indicates a narrower range of dust particle size distribution. The value of *a* represents the position of the fitted line, referred to as the fragmentation index. A larger *a* value indicates that the fitted line shifts to the left, indicating a greater production of small particle debris and a higher degree of fragmentation, i.e., a larger amount of dust generation.

**Table 3:** Experimental constants of dust Rosen-Rammler distribution after coal wall crushing under different rotational speed conditions.

Experimental constants	35 r/min	50 r/min	65 r/min	80 r/min
The degree of crushing index *a*	0.001975196	0.002462693	0.004217065	0.004899032
The crushing index *s*	1.513	1.4448	1.3053	1.2654

From the data in Table 3, it can be seen that the rotational speed of the drum cutter has a strong correlation with the crushing index *s* value and the degree of crushing index *a* value. When the drum rotation speed increases from 35 to 80 r/min, the *a* value of the coal wall after cutting and crushing increases from 0.001975196 to 0.004899032, and its *s* value decreases from 1.513 to 1.2654. With the increase in rotational speed, the degree of crushing index a value shows a gradually increasing trend, while the crushing index *s* value shows a gradually decreasing trend. This indicates that an increase in rotational speed will lead to an increase in dust generation after the drum cutter crushes the coal wall, resulting in an increase in fine dust and a more dispersed range of dust particle size distribution.

### Numerical simulation

#### Principles of discrete element force calculation between particles and drum, and among particles

To address the interaction between the coal rock particles and the cutter teeth, a linear model is used^29^. The normal contact force, tangential contact force and moment are as follows:

3 $$F_{n} = k_{n} \delta_{n} + c_{n} v_{n}$$

4 $$F_{\tau } = k_{\tau } \delta_{\tau } + c_{\tau } v_{\tau }$$

5 $$M = F_{\tau } R$$

where *k*_*n*_ and *c*_*n*_ are the normal stiffness coefficient and normal damping coefficient between the coal particles and cutter teeth, respectively; *δ*_*n*_ is the normal superposition; and *v*_*n*_ is the normal relative velocity.

**Figure 5:**
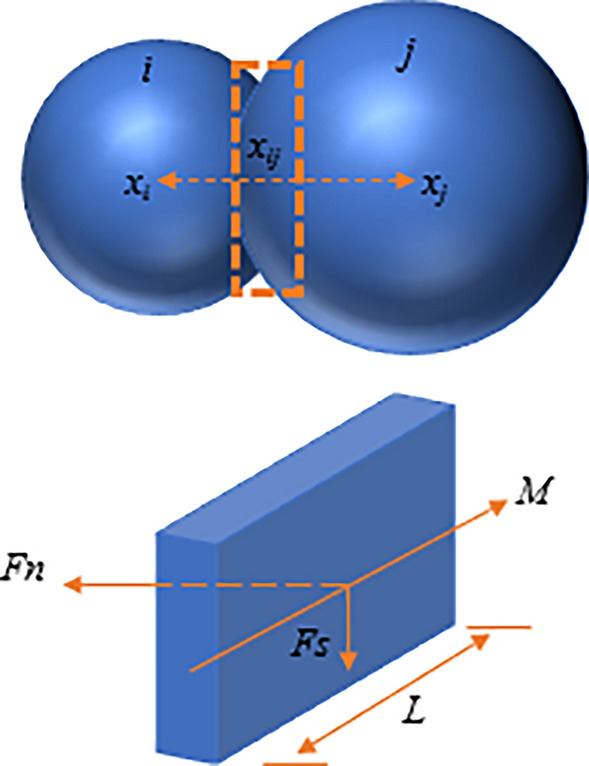
Parallel bond model between coal particles.

Considering the mechanical properties of coal, the parallel bond model^30^ is chosen in this paper to calculate the interparticle forces, and when the coal body is ruptured, the connection of the corresponding coal particles is transformed from the parallel bond model to the linear model. The two particles have a certain amount of overlap between them, forming a rectangular volume, as in Fig. 5.

The force and moment of the bond are expressed by *F* and *M*. After a certain time step of operation, the forces and moments of the bond between the two particles are as follows:

6 $$F = F + \Delta F_{n} + \Delta F_{s}$$

7 $$M = M + \Delta M$$

where Δ*F*_*n*_ is the incremental normal force after running a certain time step, Δ*Fs* is the incremental tangential force after running a certain time step, and $\Delta M$ is the incremental moment after running a certain time step.

#### Calculation principle of the motion law

**Figure 6:**
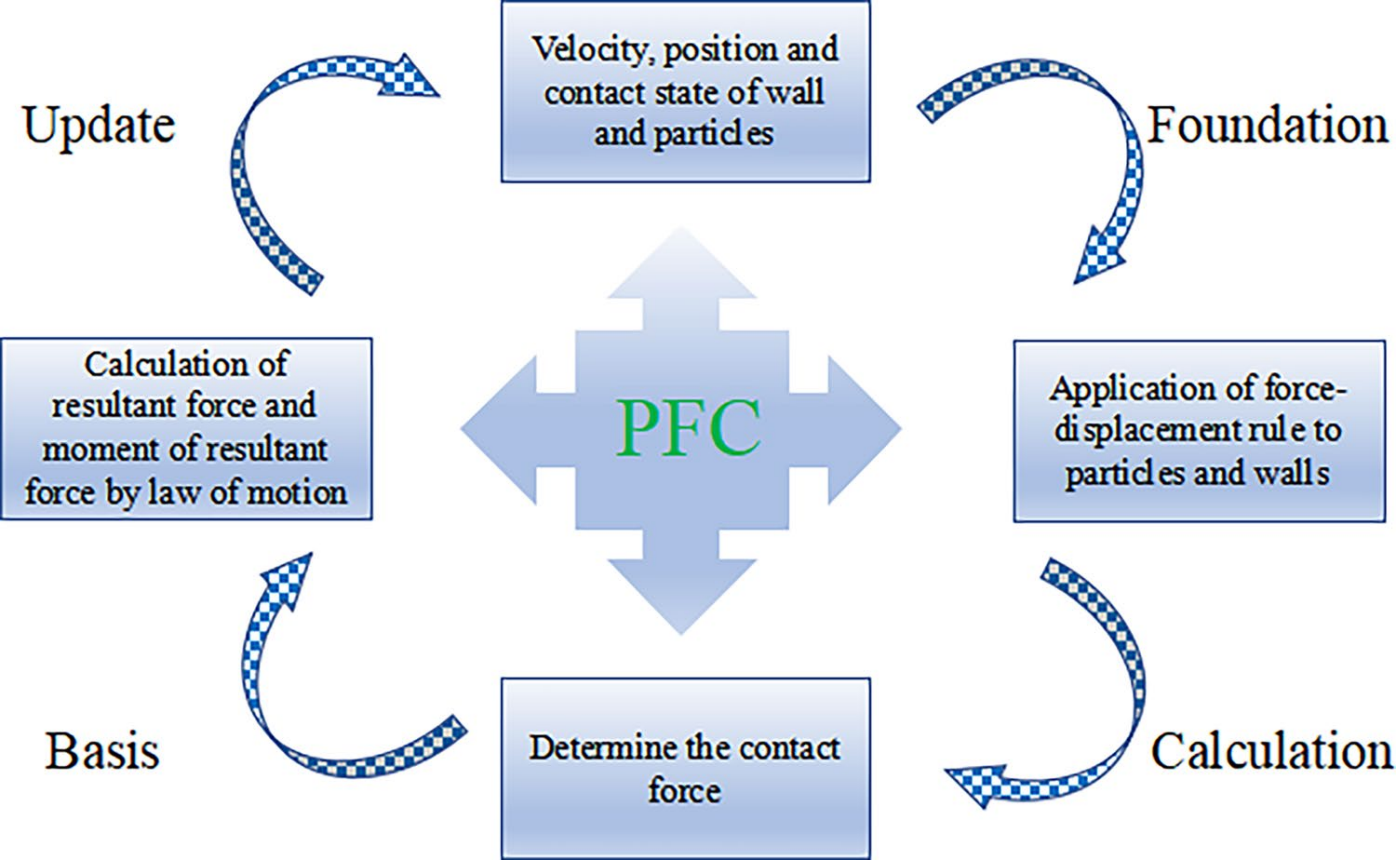
PFC operation calculation process.

The PFC calculation principle is based on the contact constitutive model, Newton’s second law of motion, and the force‒displacement law^31^, which is solved iteratively to derive the motion process of the particles, as shown in Fig. 6. First, the velocity and position of the wall and the particles as well as the contact state are determined, then the contact forces between the particles and with the wall are calculated, the forces and moments applied to each particle are found, the positions of the particles and the wall at the next calculation step are determined, the information related to the particles, the wall, and the contact is updated, and the calculation is performed again for the next cycle.

#### Calibration of model parameters

**Figure 7:**
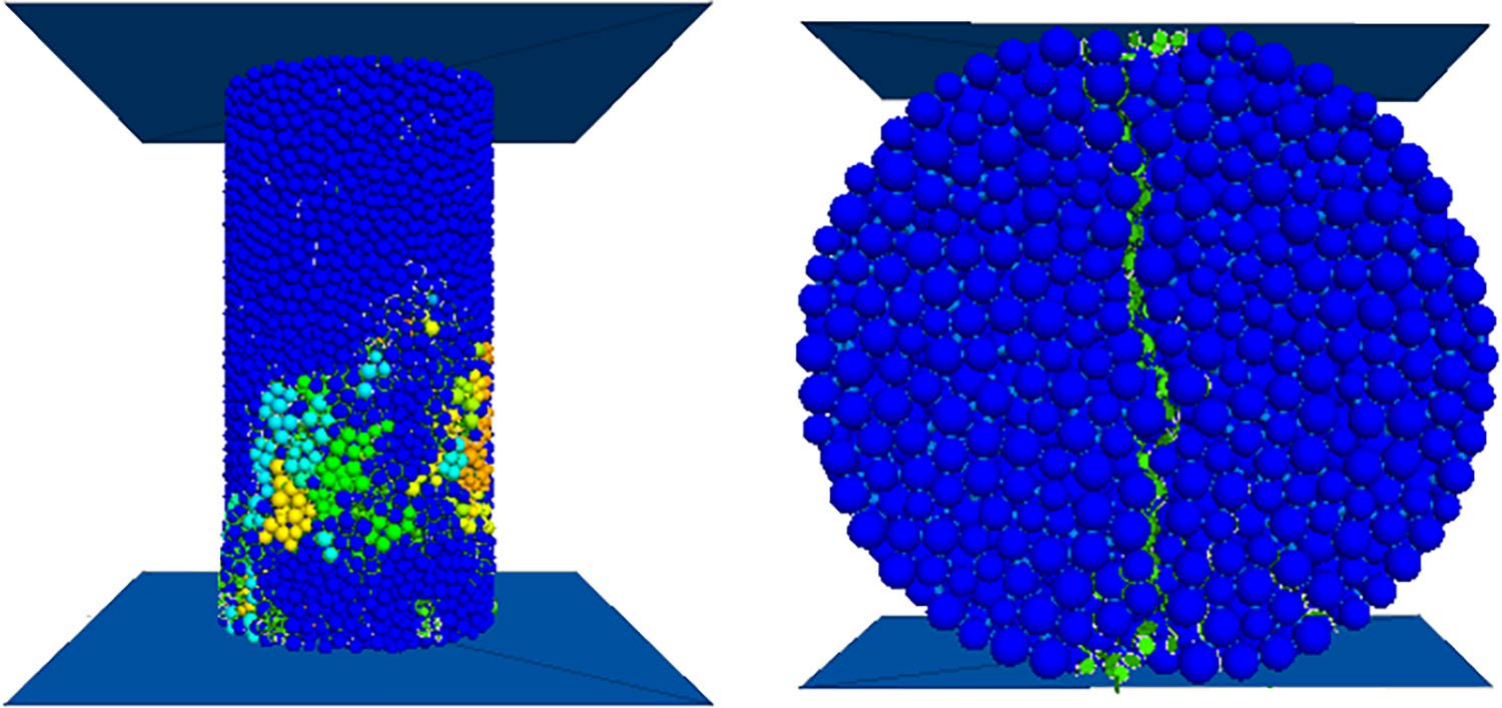
PFC^3D^ simulation results of BTS and UCS experiments.

**Figure 8:**
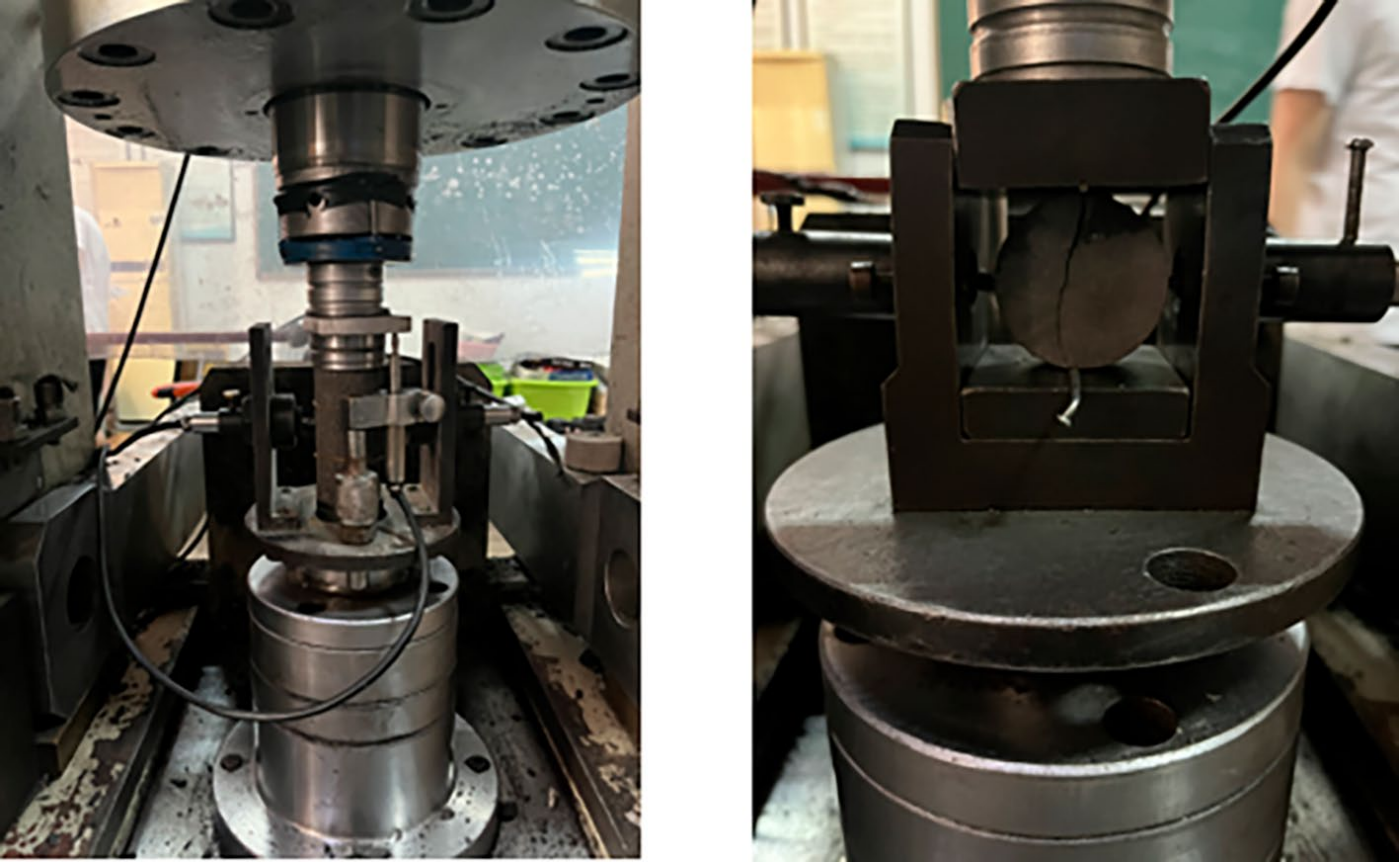
Uniaxial compression (UCS) and Brazil splitting (BTS) experiment.

The micro-mechanical parameters of the model are repeatedly modified using the trial and error method until the macroscopic mechanical parameters of the simulated material match the laboratory measurement results. The micro parameters are calibrated through uniaxial compression (UCS) and Brazilian tensile (BTS) experiments, as shown in Figs. 7 and 8. The UCS and BTS experimental models are cylindrical specimens with aspect ratios of 2:1 and 1:2, respectively. The dimensions of the UCS sample are a height of 100 mm and a diameter of 50 mm, while the dimensions of the BTS sample are a height of 25 mm and a diameter of 50 mm. The calibration results are shown in Table 4, and the micro-mechanical parameter settings are shown in Table 5.

**Table 4:** Laboratory mechanical test and PFC calibration results.

Macroscopic characteristics	Elastic modulus (GPa)	Compressive strength (MPa)	Tensile strength (MPa)
Physical experiment	1.56	9.42	1.69
Numerical simulation	1.54	9.41	1.66

**Table 5:** Set values of mesoscale parameters.

Bonded elastic modulus/GPa	Bonded stiffness ratio	Friction coefficient	Bonded tensile strength/MPa	Cohesion/MPa	Friction angle/°
2.48	7.29	0.53	3.14	1.44	26.45

#### Establishment of the simulation model of roller cutting coal wall

**Figure 9:**
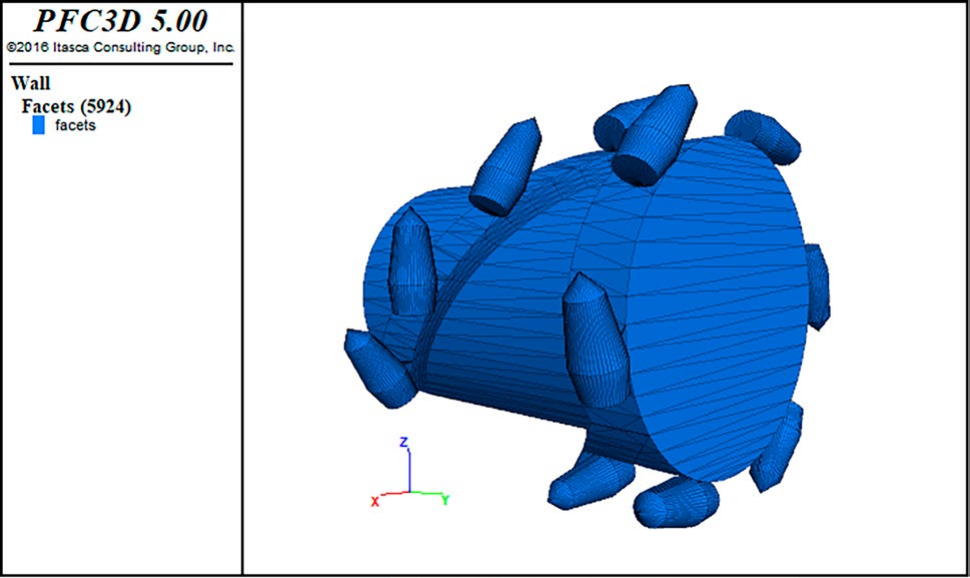
Drum model.

The article is based on a similar experiment of drum cutting of coal walls and establishes a coal wall model with dimensions of 1.5 m × 0.6 m × 1.3 m. Using the drum from the similar experiment as the research carrier, a 3D drum model is built using Rhino modeling software and imported into PFC to integrate non-graphical information and geometric topology information, as shown in Fig. 9. The spatial movement of the drum can be regarded as a composite motion of translation and rotation, with the drum’s horizontal movement direction being the negative x-axis direction and rotating counterclockwise.

**Figure 10:**
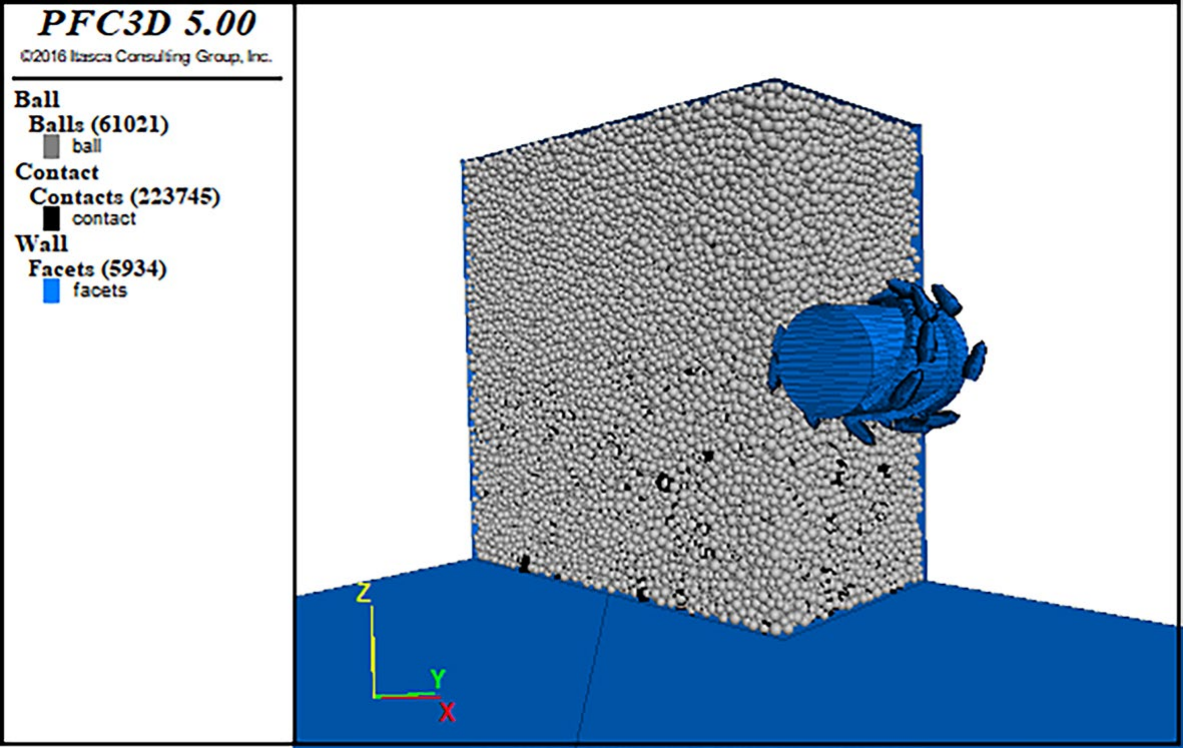
Model of roller cutting coal wall.

Since the dust production characteristics of coal wall cutting by the shearer are the focus of this simulation study, the particle size should match the actual dust particle size. However, current computers cannot meet the computational requirements, so the particle diameter needs to be enlarged. The movement of free particles reflects the ideal state of coal and dust production without the influence of airflow. The generated particle sizes range from a minimum radius of 10 mm to a maximum radius of 15 mm, totaling 61,021 small spherical particles and 223,745 bonds. The wall general and other Fish languages are used to generate wall structures to represent the uncut parts of the coal wall and the ground, which are simplified wall models that appear in actual working conditions. The upper, lower, left, and rear sides of the coal wall are fixed to prevent the simulated collapse of the coal wall during cutting. The traction speed of the drum is set to 20 mm/s, the cutting depth is 25 cm, and the time step is 2e − 6 s. The process of simulating the cutting of the coal wall by the drum until the drum completely penetrates the coal wall is carried out for drum speeds of 35, 50, 65, and 80 r/min, as shown in Fig. 10.

## Analysis of results

### Analysis of the coal wall crushing and crack evolution process

**Figure 11:**
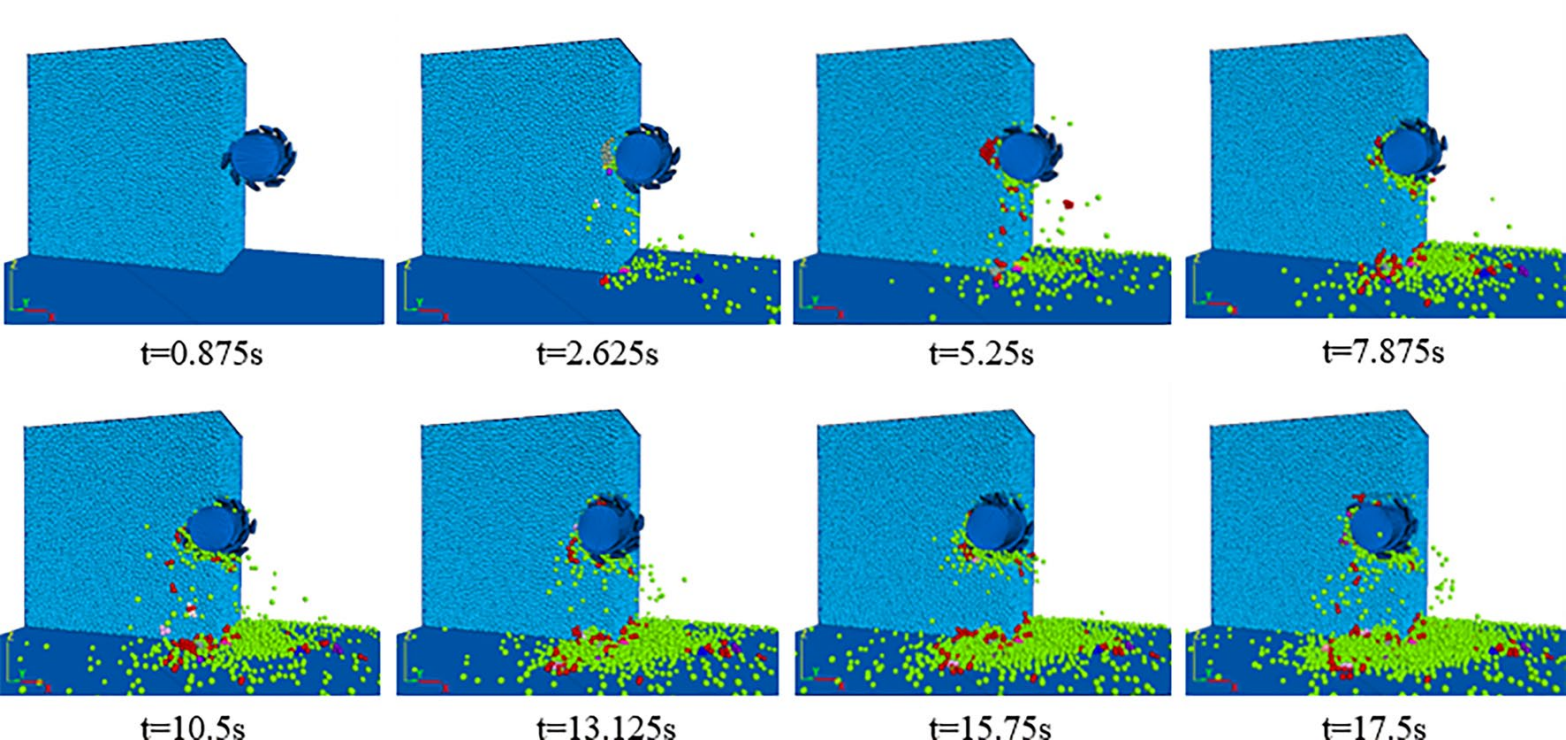
Simulation diagram of the coal wall cutting process by roller.

The entire process of roller intrusion into the coal wall takes a total of 17.5 s, and the eight moments of 0.875 s, 2.625 s, 5.25 s, 7.875 s, 10.5 s, 13.125 s, 15.75 s and 17.5 s are selected for further observation, as shown in Fig. 11.

As shown in Fig. 11, when the roller first contacts the coal wall, the cutter teeth start to apply forces to the coal body, and after reaching the stress limit of the coal body, tensile shear damage starts to occur inside the coal body, forming a fracture zone and generating a coal block. Because the contact area between the tooth tip and the coal block is small at this time, the fracture zone formed is also relatively small, and fewer coal particles are ejected. As the coal mining work proceeds, the roller goes deeper and deeper into the coal body, the diameter of roller entering the coal wall increases, the number of cutter teeth wedged into the coal body increases, the cut-off force increases, the contact stress also further increases, and many coal particles flake off from the coal wall but cannot be discharged in time due to the tightness of the cutter teeth arrangement. As the roller penetrates deeper into the coal body, the cut-off force increases and the energy accumulates, causing many coal blocks to crumble off the coal wall. At this time, a large amount of dust is rapidly released from the coal wall, resulting in a high concentration of dust in the coal mining face. When the roller fully works within the coal wall, the contact area is basically constant at this time, and the amount of dust production tends to be stabilize.

**Figure 12:**
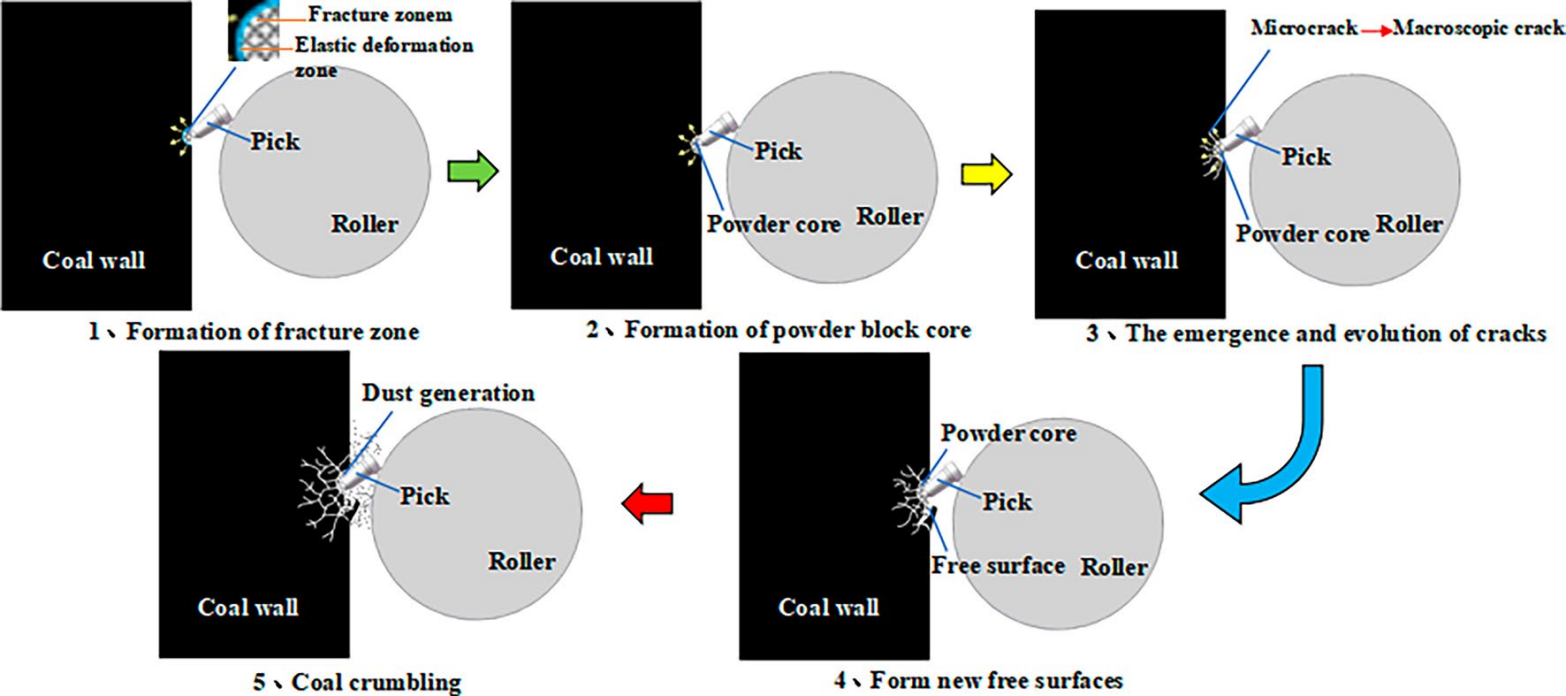
Dust production process of roller cutting coal wall.

The dust production process of coal wall cutting by a roller can be divided into five steps: formation of a crushing zone, formation of the powder block nucleus, formation and expansion of cracks, formation of new free surfaces and crumbling of the coal rock, as shown in Fig. 12. When a tooth tip just touches and squeezes the surface of the coal wall, it will produce a small elastic deformation, but the compressive stress and tensile stress applied to the coal body will produce plastic change after they are larger than the coal body’s compressive and tensile strengths, forming a crushing zone. During the process of coal wall being crushed and squeezed, fine particles are generated, and a lot of dust is also produced by the friction and collision between the cutting teeth and the coal wall. The dust formed at this time gathers in the powder block nucleus. When the cutter teeth penetrate the coal body to a certain depth, the stress and energy transferred by the powder block nucleus exceeds the strength of the coal body, and microcracks start to form at the junction of the powder block nucleus and the elastic zone of the unbroken coal body. After the macroscopic cracks expand outwards and become larger cracks, the surface of the coal body where the cracks are located is exposed to air and thus is a new free surface. With the formation of many new free surfaces, many large pieces of coal on the coal wall crumble. At this time, the stress constraint of the powder block nucleus is eliminated, and the accumulated energy releases many very small powder particles in the powder block nucleus into the air to form dust.

## Validation of the discrete element model

**Figure 13:**
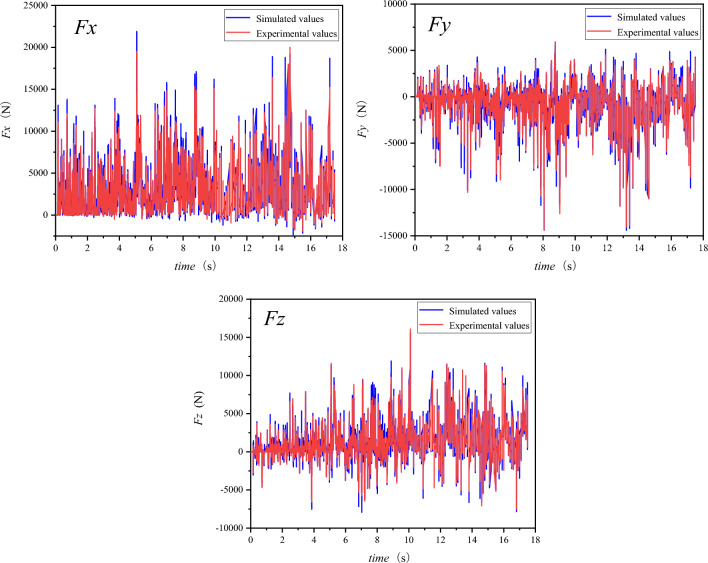
Curves of 3D forces acting on drum.

To verify the reliability of the drum cutting model, the simulation process recorded the x, y, and z three-axis forces. Figure 13 shows the comparison between the simulated results of the three-axis forces of the drum and the experimental results when the traction speed is 20 mm/s, the rotational speed is 50 r/min, and the cutting depth is 25 cm. From the figure, it can be observed that the simulated results of the drum’s three-axis forces are close to the experimental results in terms of fluctuation amplitude and trend. Therefore, the discrete element model of drum cutting coal wall established above has a certain degree of reliability in simulating the coal wall crushing process.

## Analysis of coal body fragmentation and dust generation characteristics

**Figure 14:**
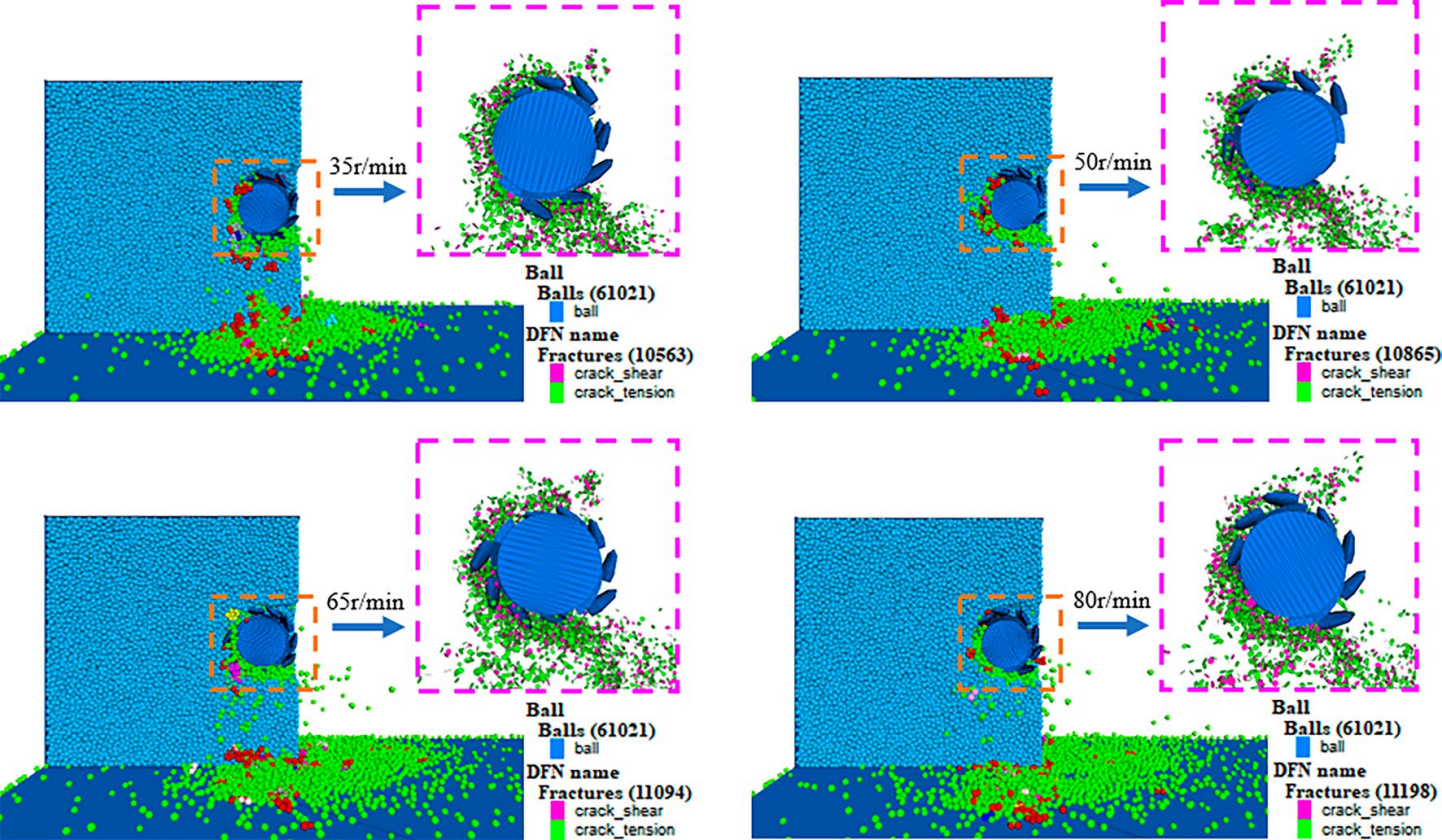
Dust production simulation diagram of lignite cutting under different rotational speeds conditions.

Figure 14 depicts the simulation results of coal cutting and crushing under different drum rotation speeds. The debris generated from cutting is represented by small colored spheres, with shear cracks and tensile cracks denoted by pink and green circular pieces, respectively. Analyzing the influence of drum rotation speed on coal fragmentation and dust generation involves examining the morphology of coal fragmentation, the development of cracks, debris volume, and the number of free single particles. Upon examining the results of coal wall cutting and crushing at four different rotation speeds, it is evident that the direction of crack propagation is generally consistent. The cracks are all propagated from the drum position to the surrounding coal wall.

**Figure 15:**
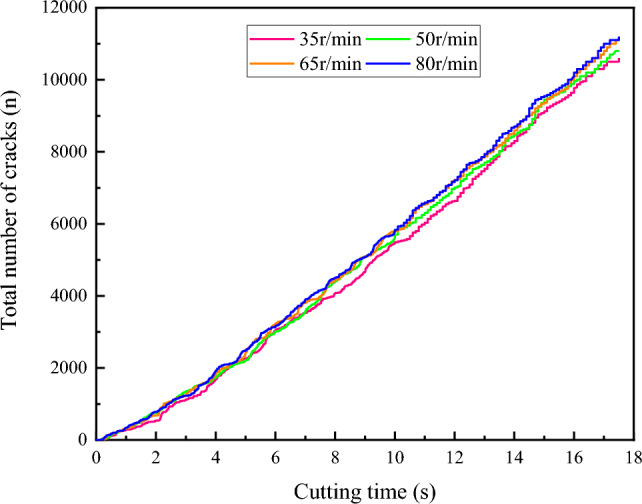
Variation trend of lignite crack number with cutting distance under different rotational speeds.

A comparison chart of the evolution of the total number of coal body cracks under different drum rotation speeds is shown in Fig. 15. From the chart, it can be observed that the growth patterns of the total crack numbers under the four rotation speed conditions are similar, all exhibiting a fluctuating growth trend. The development trends of the crack numbers at different rotation speeds are similar, all experiencing a sharp increase at a certain moment. This is due to the sharp increase in contact stress and cutting resistance as the drum continues to invade, resulting in the generation of numerous cracks. After the cracks penetrate, the coal blocks peel off, and the growth rate of the cracks slows down or even ceases. The sharp growth periods of crack numbers under different rotation speed conditions vary, occurring at different times. This is because, under the same drum structure and other motion parameters, the number of tooth invasions and cutting capabilities of the coal body differ at different rotation speeds, leading to different numbers of tooth invasions into the coal body at the same moment.

**Figure 16:**
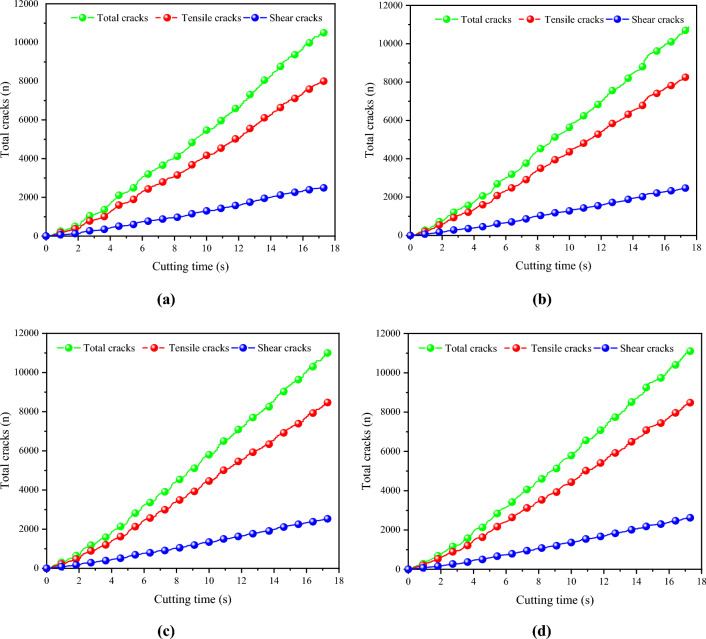
The number of various cracks developed with time at different rotational speeds.

Figure 16 depicts the development of the number of different types of cracks over time at various drum rotation speeds. In the figure, the green line represents the total number of cracks, the red line represents the tensile cracks, and the blue line represents the shear cracks. The numbers and proportions of different types of cracks under the four drum rotation speeds are shown in Table 6. It can be observed that the development trends of cracks at different speeds are basically the same, and the expansion patterns of different types of cracks at the same speed are extremely consistent. When the rotation speed is 80 r/min, the highest number of cracks is produced, reaching 11,200. As the speed decreases, the number of cracks generated by the coal wall gradually decreases, with the lowest number of cracks produced at a speed of 35 r/min, totaling 10,600. Tensile cracks account for a large proportion of the cracks generated by the coal wall under the four conditions, exceeding 76% of the total number of cracks, with a small number of shear cracks formed in each case.

**Table 6:** Number and proportion of different types of cracks.

Rotational speed of roller (r/min)	Total number of cracks	Number of tensile cracks	Proportion of tensile cracks	Number of shear cracks	Proportion of shear cracks
35	10,600	8060	77.08	2510	22.92
50	10,864	8357	76.95	2507	23.05
65	11,100	8550	76.39	2540	23.61
80	11,200	8550	76.27	2640	23.73

The volume of fragments and the number of free single particles generated by cutting the coal wall model were also calculated using programming language in PFC-3D, and the results are summarized in Table 7.

**Table 7:** Lignite chip volume and free single particle number under different rotation speeds.

Rotation speed (r/min)	Fragment volume (m^3^)	Number of free single particles
35	0.021607	2555
50	0.0222312	2627
65	0.0222488	2632
80	0.023024	2728

From Table 7, it is evident that as the drum rotation speed increases from 35 to 80 r/min, the volume of debris generated from coal wall cutting and crushing increases from 0.021607 to 0.023024 m^3^. The volume of debris is positively correlated with the drum speed, while the roughness index obtained in a similar experiment is negatively correlated with the drum speed. However, both relationships imply that the degree of coal wall fragmentation increases with the increase of drum rotation speed. The number of free single particles generated by the discrete element coal wall model increased from 2555 to 2728, and the mass ratio of dust generated in the similar experiment increased from 0.46 to 0.58%. The consistent changes in both models indicate that the cutting dust production increases with the increase of drum speed. The conclusions obtained from the discrete element simulation and the similar experiment are highly consistent, confirming the correctness of both models. In actual underground production processes, an increase in drum speed will lead to increased dust generation, resulting in reduced visibility at the work site. This phenomenon is caused by the secondary collision and fragmentation of the coal blocks cut by the drum and the drum cutting teeth. The fragmented coal blocks cannot be promptly removed, increasing the probability of secondary or multiple collisions between fragmented coal blocks and cutting teeth, thereby increasing dust generation. The increase in drum speed leads to an increase in the number of cutting teeth involved in the coal wall crushing process within the same time period, resulting in increased input energy, which in turn leads to greater coal wall fragmentation and higher dust generation.

## Conclusion

1. Drum cutting and crushing experiment were conducted at four drum speeds of 35 r/min, 50 r/min, 65 r/min, and 80 r/min. It was found that as the speed increased from 35 to 80 r/min, the roughness index decreased from 641.2 to 618.44, the mass proportion of dust with a particle size below 75 μm increased from 0.46 to 0.67%, the crushing index “a” increased from 0.001975196 to 0.004899032, and the dispersion index “s” decreased from 1.513 to 1.2654. This indicates that as the drum speed increases, the coal wall is more severely crushed, the amount of dust generated increases, the particle size distribution range becomes wider, and the dust particles generated become finer.
2. A discrete element model of coal wall cutting and crushing at a scale of 1:1 was established based on the similar experimental platform. The simulation found that the drum speed has little effect on the direction of internal crack propagation and type of damage in the coal wall. The cracks are all propagated from the drum position to the surrounding coal wall, mainly in tension failure, and the tensile cracks account for more than 79% of the total cracks.
3. As the drum speed increased from 35 to 80 r/min, the volume of debris generated by the discrete element model of the broken coal wall increased from 0.021607 to 0.023024 m^3^, and the number of free individual particles increased from 2555 to 2728. This indicates that the degree of damage and the amount of dust generation are positively correlated with the drum speed, and both increase with increasing speed. This is highly consistent with the experimental conclusions, proving the correctness of the simulation and experiment. Therefore, controlling the drum speed reasonably in the comprehensive mining face can effectively reduce dust pollution from the source.

## Data Availability

Data is provided within the manuscript.
